# Optical anisotropy enables skyrmions for robust information encoding

**DOI:** 10.1038/s41377-026-02396-1

**Published:** 2026-07-17

**Authors:** Ziyu Zhan, Qiang Liu, Xing Fu

**Affiliations:** 1 Department of Precision Instrument, Tsinghua University, Beijing, China; 2 State Key Laboratory of Precision Space-Time Information Sensing Technology, Beijing, China; 3 Key Laboratory of Photonic Control Technology (Tsinghua University), Ministry of Education, Beijing, China

**Keywords:** Optical physics, Optical physics

## Abstract

Skyrmions are typically tied to sphere-valued vector fields. Here, researchers break this paradigm by generalizing their construction through parameter space dimensionality reduction and realizing it in structured optical anisotropy, which enables directly readable and perturbation robust skyrmionic states for topological information encoding.

Originally introduced in particle physics^1^ and later made famous in magnetism^2^, skyrmions have emerged as a unifying concept across diverse branches of wave and matter physics. Optical, acoustic and hydrodynamic systems can all host skyrmionic textures once an appropriate field is mapped onto an effective two-sphere^3–5^. Despite this versatility, most existing realizations still depend, either explicitly or implicitly, on fields that are naturally constrained to such sphere‑valued descriptions. This dependence imposes a fundamental constraint on the accessible topological design space and, in turn, restricts the practical utility of skyrmions for information technologies. The limitations are particularly pronounced in the context of information encoding. Magnetic skyrmions are compact but sensitive to material and thermal conditions^6^. Optical skyrmions are comparatively easy to generate and manipulate, but are more naturally suited to transfer than storage^7^. Liquid crystal skyrmions offer an appealing structured matter platform, but conventional implementations still face restrictions in data density, readout flexibility and the range of reliably sustainable topological states^8^. Therefore, the broader challenge is not simply how to create another skyrmion platform, but how to harness higher dimensional physical degrees of freedom for robust, information-rich topological encoding.

Recently^9^, Zhang and co-workers address this challenge by shifting the focus from real-space vector fields alone to the high-dimensional optical response of structured matter. Their key idea is that skyrmionic topology need not reside in the full physical field itself, but can emerge from a reduced, physically meaningful subspace. To formalize this concept, they introduce a framework termed parameter space dimensionality reduction, in which a high dimensional field is projected onto a sphere-valued field. Importantly, rather than merely a mathematical reformulation, the framework finds concrete realization in an experimentally accessible quantity: the optical anisotropy of spatially varying retarders.

Specifically, the authors identify the axis geometry embedded within the Mueller matrix description of a structured anisotropic medium. This axis geometry field provides an effective sphere-valued map from which skyrmions can be defined. In this way, the topology is encoded not in a conventional free-space optical field or a standard matter director field, but in the anisotropy geometry of structured matter itself. Thus the resulting objects, termed axis-geometry-based (AGB) skyrmions, constitute a distinct skyrmion family rooted in the vectorial optical response of the medium. The concept is experimentally realized using a tunable elliptical retarder array synthesized from cascaded liquid crystal spatial light modulators. This platform enables pixel-level programming of spatially varying anisotropy, while Mueller matrix polarimetry and decomposition provide direct reconstruction of the resulting topological states. The experimental results reveal a rich family of topological textures, including Néel-type skyrmions, Bloch-type skyrmions, higher-order skyrmions, skyrmion bags, skyrmion lattices and meron lattices. This diversity is itself significant, showing that once topology is engineered in a high dimensional anisotropy space instead of a conventional low dimensional field, the accessible skyrmion landscape expands substantially.

Beyond generation, the work systematically addresses a question of central practical importance: how robust are these topological states against disorder? By introducing controlled Gaussian perturbations, the authors track the evolution of the skyrmion number as the perturbation magnitude increases. They identify three distinct regimes: a stability regime in which the topological charge remains preserved, a transition region characterized by gradual degradation, and a collapse regime at high perturbation levels where topology is ultimately lost. This behaviour is consistent with a sufficient criterion for topological protection derived in the paper, formulated as the 60° rule. In practical terms, this rule provides an intuitive engineering guideline: if the realized field deviates locally from the target field by no more than 60°, the topology remains protected. The emergence of such a simple criterion from a high dimensional topological framework is both conceptually elegant and practically useful. The practical significance of this robustness is highlighted in the final demonstration, where the authors use skyrmion bags for topological information storage. By assigning skyrmion numbers to constituent structures within a skyrmion bag, they encode ASCII information and recover it experimentally even in the presence of perturbation. This proof-of-concept is more than illustrative. It shows that the proposed framework is not merely a new language for classifying topological textures, but a viable route towards physically accessible, optically readable and perturbation-resistant information encoding (see Fig. 1).

**Fig. 1:**
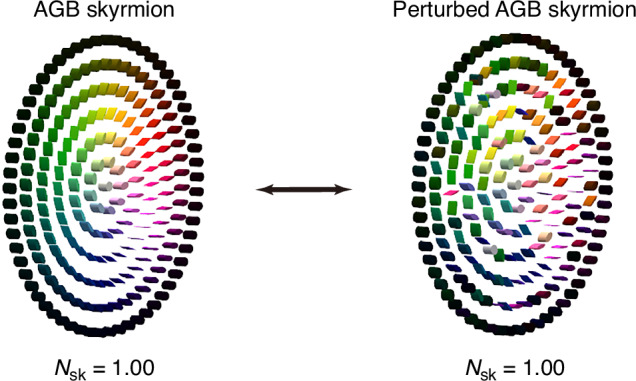
Schematic illustration of an AGB skyrmion encoded in the anisotropy axis geometry of structured matter, showing its robustness to perturbations conferred by topological protection. N_sk_ represents skyrmion number

Furthermore, this work signals a paradigm shift in how skyrmions may be designed in photonic and structured matter systems. Instead of asking whether a field is naturally sphere-valued, one can ask whether a physically meaningful sphere-valued subspace can be identified within a higher dimensional response. This shift opens a wider design space and aligns naturally with ongoing efforts in vectorial optics, structured matter and topological structured light, where relevant information is often distributed across coupled degrees of freedom. However, a key challenge remains that the present implementation relies on a synthetic architecture built from cascaded bulky retarder elements, as opposed to a single compact platform capable of directly realizing arbitrary elliptical retarders. This limitation is technological rather than conceptual, but it will matter for future deployment. Progress in metasurfaces^10^, laser-written birefringent media^11^ and reconfigurable retarder platforms^12^ may therefore be crucial for translating this framework into scalable devices. In summary, this work establishes optical anisotropy as a new host for skyrmionic topology, offering a promising route toward robust topological information encoding in structured matter.
